# Socioeconomic and demographic factors predictive of missed appointments in outpatient radiation oncology: an evaluation of access

**DOI:** 10.3389/frhs.2023.1288329

**Published:** 2023-11-27

**Authors:** Allen M. Chen

**Affiliations:** ^1^ Department of Radiation Oncology, University of California, Irvine, Irvine, CA, United States; ^2^ Chao Family Comprehensive Cancer Center, Orange, CA, United States

**Keywords:** radiation oncology, scheduling, disparities, access, missed appointments

## Abstract

**Purpose:**

While missed patient appointments reduce clinic efficiency and limit effective resource allocation, factors predictive of “no shows” are poorly understood in radiation oncology.

**Methods and materials:**

A prospective data registry of consecutive patients referred for initial consultation from October 2,018 to April 2022 was reviewed. Demographic characteristics recorded included age, gender, race, language preference, living situation, and insurance status. Zip code data linked to a patient’s residential address was used to determine socioeconomic status (SES) based on publicly available data on median household income. No show encounters were defined as all encounters where the patient failed to cancel their visit and did not sign-in to their scheduled appointment. Descriptive statistics were presented to identify factors predictive of missed appointments.

**Results:**

A total of 9,241 consecutive patients were referred and logged into the database during the 4-year period, of which 5,755 were successfully scheduled and registered. A total of 523 patients (9%) failed to show for their appointments. Missed appointments were associated with low-income status, homeless living situation, and Black or Latino race (*p* < 0.05, for all). The proportion of White, Latino, Asian, and Black patients who missed appointments was 6%, 14%, 9%, and 12%, respectively (*p* < 0.001). Patient characteristics independently associated with higher odds of appointment non-adherence included low-income status ((OR) = 2.90, 95% CI (1.44–5.89) and Black or Latino race [(OR) = 3.31, 95% CI: 1.22–7.65].

**Conclusions:**

Our results highlight the influence of demographic, financial, and racial disparities on proper health care utilization among patients with cancer. Future interventions aimed at reducing appointment no shows could channel resources to the at risk-populations identified in this analysis, improving access to care, and optimize clinic efficiency.

## Introduction

Providing affordable, inclusive, and timely access to healthcare represents a cornerstone of any society. As health systems continue to develop methods to improve workflow efficiency, the optimization of resource utilization takes on greater importance. Missed appointments thus represent a significant barrier to quality of care as the negative effects are not just limited to the individual but have repercussions on the population as a whole. While “no-shows” have a direct impact on the continuity of patient care, they also contribute to unnecessary expense and wasteful spending given that the resources allocated and unused could have been more efficiently spent elsewhere. Indeed, the financial implications of missed appointments have been well-established in a variety of practice settings (1–3). Moreover, missed appointments also lead to wait times for other patients down the queue that could have been possibly avoided. These considerations are particularly germane in oncology care given the enormity of the costs traditionally associated with care, as well as the demonstrated importance of initiating and completing treatment in a timely fashion. A systematic review and meta-analysis of 34 studies across 7 cancer types pointedly showed that every month delay in starting treatment was associated with an approximate 10% increase in mortality (4). Notably, some of the most pronounced detrimental effects were seen in patients referred for radiation therapy. Although some investigations have attempted to identify predictors of missed appointments in healthcare, these have generally focused on primary care or non-oncology specialties (5–9). Additionally, the findings have been relatively inconsistent with age, insurance status, and ethnicity cited as possible influential factors. The purpose of this study was to therefore evaluate factors which might be predictive for missed appointments among patients referred for radiation therapy.

## Methods and materials

From October 2018 to April 2022, patients referred to the Department of Radiation Oncology at the University of California, Irvine, School of Medicine were inputted into a commercially available, enterprise-based electronic medical record system (Epic Systems, Inc. Verona, WI) for assignment to physicians for initial consultation. This information was used to prospectively populate a customized registry using data dictionaries and included fields allowing for the collection of patient-specific demographic and disease characteristics. Only patients with a known diagnosis of cancer were included. Population-based data were categorized using standard nomenclature in accordance with that determined by the United States Census Bureau (10). Race was based on self-identification and officially recognized 5 racial categories (White, Black, Asian, American Indian, Native Hawaiian/Pacific Islander). Zip code data linked to a patient’s residential address was used to determine socioeconomic status (SES) based on publicly available data on median household income (11). SES was subsequently categorized into 4 designated quartiles which correlated with income level (low, medium-low, medium-high, and high). Insurance status was classified into public versus private. Patients were deemed non-English speaking if requiring translator services including the reliance on family or other personnel for direct communication with providers.

The scheduling protocol during the time frame of the study was standardized such that each morning before 8:00am, all pre-authorized referrals collected from the prior overnight hours were compiled at a daily huddle at which time a designated intake team contacted patients via telephone for scheduling. Schedulers were instructed to leave voice messages in the event that initial contact was unsuccessful. Clinic slots were available in 1 h increments from 8am to 5pm, with one hour generally blocked for the lunch hour. Every attempt was made to accommodate the request of the patient for scheduling at a specific time. Referrals that arrived subsequently throughout the day, whether from the electronic health record system or via direct contract from referring physicians or patients were scheduled as they arrived. Once an appointment was confirmed, no separate reminders were issued to patients.

For the purpose of this study, a patient “no-show” referred to a missed patient appointment wherein the patient was scheduled, did not appear for the appointments, and made no prior contact with the clinic staff outside of 24 h from the appointment. During the time course of this study, attempts were generally made to re-schedule and/or to elucidate the reasons for the missed appointment, which were then documented in the registry. Whether or not a patient ultimately re-scheduled did not factor into the initial no-show rate. Patients who either refused consultation or informed the staff they would call back to schedule but never did were also included. To adjust for possible effects of the Covid-19 pandemic, virtual versus in-person appointments were also analyzed separately. This analysis focused specifically on new consultation appointments and thus did not include follow-up visits.

Descriptive frequencies of demographic and SES characteristics associated with patient “no-shows” were assessed. Categorical variables were compared using chi-squared statistics for frequency and proportions. Continuous variables were presented as means and compared using *t*-tests. The significance level was set at 0.05 for all analyses. To investigate correlates of appointment non-adherence, multivariable logistic regression analyses were performed assessing for age, sex, employment, insurance, and socio-economic status. Given the potential class imbalance in events across variables, standardized oversampling techniques were employed to verify the findings of the logistic regression. This study was approved by the local Institutional Review Board.

## Results

A total of 9,241 consecutive patients were referred and logged into the database during the 4-year period, of which 5,755 were successfully scheduled and registered. The remaining patients were not seen in consultation and either declined to be treated and/or seen in consultation, or alternatively, received radiation therapy elsewhere. Primary disease sites were breast (*N* = 1,399), prostate (*N* = 1,054), head/neck/skin (*N* = 759), lung (*N* = 601), gastrointestinal (*N* = 574), gynecologic (*N* = 408), central nervous system (*N* = 297), hematologic (*N* = 103), other/miscellaneous (*N* = 560). The data capture completion rate for all tabulated entries was 100%. The median age of the patients scheduled for consultation was 55 years (range, 17–101). Gender was 2,992 male (52%); 2,763 female (48%); Race was 3,165 White (55%); 1,058 Latino (18%); 1,020 Asian (18%); 461 Black (8%); and 51 Native American/Pacific Islander/Other (1%). Nine hundred and forty patients (16%) were non-English speaking. A list of all socioeconomic variable included in the analysis is outlined in Table 1.

**Table 1:** List of socioeconomic variables analyzed.

Variable
Race
Language
Socioeconomic status
Age
Insurance
Living situation
Distance from facility

A total of 112 zip codes were used to classify patients into 4 categories of 28 zip codes each to create quartiles for the purpose of analyzing SES. Using the census data, the median household incomes associated with each zip code ranged from $27,683 to $475,757 (mean, $78,570). The four created quartiles were disturbed as follows: low, below $49,999; medium-low, $50,000–$78,570; medium-high, $78,571–$101,999; high, $102,000 and above.

**Figure 1:**
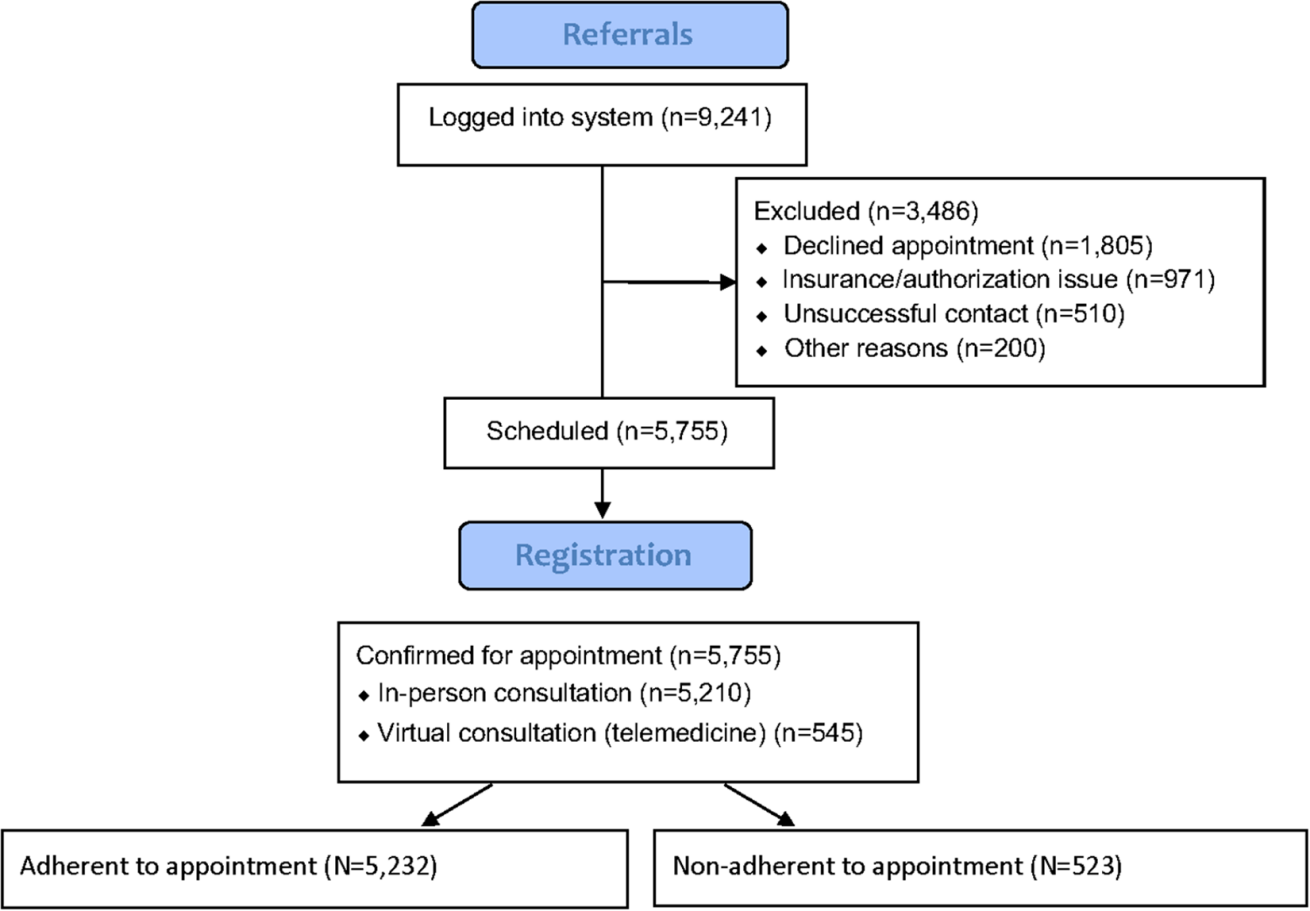
Flow diagram of patients.

Among the 5,755 patients scheduled for initial consultation, a total of 523 patients (9%) failed to show for their appointments. Among these 523 no-shows, a total of 267 patients (51%) notified the staff within 24 h prior to the scheduled appointment; the other 256 patients (49%) failed to show without notice. A total of 89 no-show patients were successfully re-scheduled among the 523 (17%). Figure 1 illustrates a flow diagram of the 9,241 patients referred. There was no difference in the missed appointment rate based on whether a patient was scheduled for a virtual or in-person appointment (*p* = 0.51). Four hundred and sixty-nine of the 5,210 patients (9%) were non-adherent to their in-person appointments compared to 54 of 545 (10%) of patients scheduled for virtual consultations (*p* = 0.49).

As illustrated in Table 2, the distribution of unadjusted patient characteristics varied across the categories of appointment keeping for those who kept and missed appointments. Missed appointments were associated with low-income status, homeless living situation, and Black or Latino race (*p* < 0.05, for all). Two hundred and two of the 1,431 patients (14%) in the lowest quartile SES were “no shows” compared to 321 of the 4,003 patients (8%) in the non-lowest quartile SES group (*p* < 0.001). Forty of 120 homeless patients (33%) missed appointments compared to 483 of the 5,222 patients (9%) who were not homeless (*p* < 0.001). The proportion of White, Latino, Asian, and Black patients who missed appointments was 6%, 14%, 9%, and 12%, respectively (*p* < 0.001). The distance from residence to facility, as measured in miles as a continuous variable, did not predict for missed appointments (*p* = 0.19).

**Table 2:** Number of patients who missed and kept their scheduled appointments.

	No shows	Shows
Race (*p* < 0.001)		
White	205 (6)	2960 (94)
Latino	152 (14)	906 (86)
Asian	91 (9)	929 (91)
Black	79 (17)	382 (83)
Other	6 (12)	45 (88)
Language (*p* = 0.06)		
English	422 (9)	4393 (91)
Non-English	101 (11)	839 (89)
SES quartile (*p* < 0.001)		
High	64 (4)	1361 (96)
Medium-high	108 (7)	1332 (93)
Medium-low	149 (10)	1310 (90)
Low	202 (14)	1229 (86)
Living situation (*p* < 0.001)		
Home	301 (8)	3707 (92)
Assisted living/nursing facility	90 (10)	812 (90)
Homeless	40 (33)	80 (67)
Other/unknown	92 (12)	703 (88)
Age (*p* = 0.51)		
≤50	160 (9)	1690 (91)
51–64	177 (9)	1751 (91)
65+	186 (9)	1791 (91)
Gender (*p* = 0.47)		
Male	280 (9)	2712 (91)
Female	243 (9)	2520 (91)
Insurance (*p* = 0.72)		
Public	300 (9)	3013 (91)
Private	223 (9)	2219 (91)

Table 3 presents results of a multivariable logistic regression analysis demonstrating the association of several factors with appointment non-adherence after adjustment for potential confounders. Patient characteristics independently associated with higher odds of appointment non-adherence included low-income status ((OR) = 2.90, 95% CI (1.44–5.89) and Black or Latino race [(OR) = 3.31, 95% CI: 1.22–7.65].

**Table 3:** Multi-variate analysis of potential factors for missed appointments.

Factor	Strata	% Non-adherence	OR	95% CI	*p*-value
Race	White	6.5%	2.55	(1.71, 9.01)	**<0** **.** **001**
	Latino	14.3%			
	Asian	8.9%			
	Black	17.1%			
	Other	11.8%			
Race	Latino/Black	15.2%	3.31	(1.22, 7.65)	**<0**.**001**
	Non-Latino/Black	7.1%			** **
Language	Non-English	8.7%	1.08	(0.55, 4.09)	0.22
	English	10.7%			
SES quartile	High	4.5%	2.21	(1.30, 6.12)	**<0**.**001**
	Medium-high	7.5%			
	Medium-low	10.2%			
	Low	14.1%			
SES	Low quartile	14.1%	2.90	(1.44, 5.89)	**<0**.**001**
	Non-Low quartile	7.4%			
Living situation	Homeless	33.3%	1.22	(0.49, 1.90)	0.09
	Non-homeless	8.5%			
Insurance	Public	9.1%	1.11	(0.53, 2.99)	0.19
	Private	9.1%			
Distance as continuous variable		–	1.30	(0.42, 2.57)	0.10
Age as continuous variable		–	0.95	(0.34, 1.12)	0.43

SES, socioeconomic status; OR, odds ratio; CI, confidence interval.

Bold values indicates statistically significant.

## Discussion

The results of the present study highlight the pronounced socio-economic and racial disparities that exist in accessing tertiary-based oncology treatment. Our findings, obtained from a prospective registry of cancer patients referred for radiation therapy, pointedly demonstrate that certain segments of the population are at higher risk for missed appointments. While the specific reasons are speculative, the implications with respect to health equity are profound given that these same groups have been consistently shown to have inferior survival from their disease.

Numerous studies have attempted to evaluate how race and ethnicity contribute to uneven access to health care (12–14). One study suggested that culturally diverse patients miss medical appointments primarily due to a perceived disrespect for their beliefs and lack of understanding of scheduling systems (12). Another showed that minority patients sometimes were unable to attend appointments with specialists due to transportation and challenges related to taking time off work (13). These data suggest that patients from underserved backgrounds often work jobs with less flexible scheduling which forces them to choose between skipping a medical appointment or work (14). More simplistically, this could also be viewed as a choice between going unpaid or making a medical appointment. Regardless of the specific reasons, the impact that social determinants of health have on access warrant continued study.

In one of the most robust studies to date, Sotudian et al. used a dataset of 9,970 patients and 36,606 to develop linear and non-linear models to identify predictive variables for missed breast imaging appointments (15). Among the 57 potentially impactful variables analyzed, the investigators found that those related to social determinants of health including housing insecurity, difficulty paying utility bills, and family caretaking were among the strongest in predicting for “no shows.”

Additionally, the concept of financial toxicity in healthcare has been increasingly identified as a powerful detriment on quality of care (16). High out-of-pocket patient costs and the potential lost income from absenteeism are well-documented care access barriers (17). From a practical standpoint, when patients cannot afford medical care or find themselves choosing between medical care and paying for other utilities like rent, mortgage, or food, they often go without healthcare access. Financial toxicity is especially relevant in radiation oncology due to rising costs associated with expensive technology and the weeks required for treatment. Studies have shown that with a diagnosis of cancer, the risk of bankruptcy and/or home foreclosure increases significantly (18).

Although insurance status was not shown to be predictive of missed appointments, its role in enabling access to health care and to protect families from high medical costs is also germane as patients from disadvantaged backgrounds have faced longstanding disparities in health coverage that contributes to differing barriers to access. While the Affordable Care Act created new health coverage options that helped to narrow these disparities, studies have shown that Black and Latino individuals continued to lag with respect to health insurance coverage compared to their Caucasian counterparts (19). The higher rates of uninsured among these groups largely reflects more limited rates of private coverage among these groups which was consistent with what was seen. While Medicaid expansion helped fill the gap in private coverage for people of color, they do not fully offset the difference (20).

On the most basic level, when patients miss appointments, continuity of care is interrupted; as a result, cancers might not be effectively monitored nor treated, and the risk of complications from neglect increases. Additionally, the phenomenon of up-staging (i.e., progression of disease to more advanced stages) has been well-described and is one reason why underrepresented minorities may have higher mortality rates from cancer (21). Studies have shown that even short delays in initiating treatment for cancer adversely affects survival (21–23). From a radiobiological standpoint, tumor cells have the potential to grow into more hypoxic and resistant phenotypes with delay (24). When a patient ultimately then decides to initiate treatment, more resources may then be required. For instance, a patient who may have been potentially curable with radiation therapy alone now may require higher doses of treatment as well as possible chemotherapy. Additionally, patients sometimes will then use the emergency room as their point of entry into oncology care (25).

It’s also important to realize that every missed appointment causes logistical issues for the healthcare system. While the absent patients see their condition worsen, those absences simultaneously cause a delay in care for others. The result is a massive underutilization of resources leading to increased expenses. Indeed, much has been made about the economic effects of patient “no-shows” on the health care system (26–28). Data has shown that missed appointments cost the US health care system more than $150 billion a year and individual physicians an average of $200 per unused time slot (29, 30). Given the vast expense of maintaining and operating a radiation oncology practice, underutilization of resources is relevant.

The human element cannot be underestimated. Medical appointments can routinely conjure up emotions of fear and despair that can be exacerbated in certain underserved communities. Studies have shown that the high no-show rates with some medical services might be due to anxiety and perceived discomfort, particularly with the onset of the pandemic (31). A lack of trust, which can compound the fear of having to navigate a complex system has also been shown to be prevalent among the underrepresented. For instance, the sentiment that the health system is designed to serve White individuals and is not as welcoming to underrepresented populations still exists (32). In our multivariate model, we found that race even surpassed SES as the most significant social determinant which predicted for missed appointments, suggesting that deeply rooted, historic causes related to culture may underlie these findings.

One of the major limitations of this paper is that the exact reasons for “no-shows” could not be determined. While follow-up phone calls were routinely placed to patients who missed appointments, the explanations often could not be extracted and/or were not documented. The reliance on zip code data to define SES was also imperfect and could misrepresent a patient’s actual income status and/or educational level. The latter is especially important as studies have consistently shown that access improves as one’s education rises. Further, we were unable to show how these access disparities affected such endpoints related to cancer outcomes. Others, however, have documented the link between access and survival (33). Finally, given the relatively limited size of this analysis, our goal was not to develop a predictive model for clinical use but rather to engage in hypothesis generation. Future work will focus on the construction of a practical framework which may have operational utility. Additionally, qualitative research methods might be suited to understand the reasons for missed appointments with more granularity such that interventions could be developed to improve patient compliance.

The results of the present series are particularly instructive because they illustrate how social determinants of health potentially impact access to radiation oncology services. Notably, significant barriers were identified for patients of underrepresented ethnic backgrounds and lower income in keeping appointments for higher-level oncology care. While the reasons remain speculative, efforts to ensure that care is equitable and culturally competent must be improved so that the playing field for disadvantaged communities is levelled. This will require engagement from all stakeholders and the appropriate resource allocation to address issues related to transportation, employment, and coverage, among others. Previous work has suggested that such initiatives as open access scheduling, extended clinic hours, and electronic communication have the potential to improve compliance to outpatient appointments (34). The development of multi-disciplinary clinics, where patients can see multiple providers simultaneously, has also been shown to enhance coordination and lead to less delays in initiating care (35, 36). Several studies also have recently demonstrated how artificial intelligence could be useful to identify those at risk for missed appointments and potentially establish targeted strategies for improvement (37, 38). Regardless, community-based, culturally tailored educational programs including the development of financial navigation and patient-assistance programs will be important to address the challenges facing vulnerable populations.

## Conclusions

The influence of demographic, financial, and racial disparities on proper health care utilization among patients with cancer is significant. Future interventions aimed at reducing appointment no shows could channel resources to the at risk-populations identified in this analysis, improving access to care, and optimize clinic efficiency.

## Data Availability

The raw data supporting the conclusions of this article will be made available by the authors, without undue reservation.

## References

[1] Huang Z Ashraf M Gordish-Dressman H Mudd P. The financial impact of clinic no-show rates in an academic pediatric otolaryngology practice. Am J Otolaryngol. (2017) 38(2):127–9. 10.1016/j.amjoto.2016.11.004 27913067

[2] Moore CG Wilson-Witherspoon P Probst JC. Time and money: effects of no-shows at a family practice residency clinic. Fam Med. (2001) 33:522–7. 11456244

[3] Mieloszyk RJ Rosenbaum JI Hall CS Raghavan UN Bhargava P. The financial burden of missed appointments: uncaptured revenue due to outpatient no-shows in radiology. Curr Probl Diagn Radiol. (2018) 47:285–6. 10.1067/j.cpradiol.2018.06.001 29929841

[4] Hanna TP King WD Thibodeau S Jalink M Paulin GA Harvey-Jones E Mortality due to cancer treatment delay: systematic review and meta-analysis. Br Med J. (2020) 371:1–11. 10.1136/bmj.m4087 PMC7610021 33148535

[5] Kaplan-Lewis E Percac-Lima S. No-show to primary care appointments: why patients do not come. J Prim Care Community Health. (2013) 4:251–5. 10.1177/2150131913498513 24327664

[6] Lee VJ Earnest A Chen MI Krishnan B. Predictors of failed attendances in a multi-specialty outpatient centre using electronic databases. BMC Health Serv Res. (2005) 5:51. 10.1186/1472-6963-5-51 16083504 PMC1190171

[7] Rosenbaum JI Mieloszyk RJ Hall CS Hippe DS Gunn ML Bhargava P. Understanding why patients no-show: observations of 2.9 million outpatient imaging visits over 16 years. J Am Coll Radiol. (2018) 15:944–50. 10.1016/j.jacr.2018.03.053 29755001

[8] Fiorillo CE Hughes AL I-Chen C Westgate PM Gal TJ Bush ML Factors associated with patient no-show rates in an academic otolaryngology practice: no-show rates in an otolaryngology practice. Laryngoscope. (2018) 128:626–31. 10.1002/lary.26816 28815608 PMC5814324

[9] Ellis DA McQueenie R McConnachie A Wilson P Williamson AE. Demographic and practice factors predicting repeated non-attendance in primary care: a national retrospective cohort analysis. Lancet Public Health. (2017) 2:551–5559. 10.1016/S2468-2667(17)30217-7 PMC5725414 29253440

[10] United States Census Bureau. *Income data tables*. (2022). Available at: https://www.census.gov/topics/income-poverty/income/data/tables/acs.html (Accessed January 1, 2023).

[11] California Open Data Portal. *Income tax statistics by zip code*. Available at: https://data.ca.gov/dataset/personal-income-tax-statistics-by-zip-code (Accessed January 1, 2023).

[12] Shavers VL Brown ML. Racial and ethnic disparities in the receipt of cancer treatment. J Natl Cancer Institut. (2002) 94:334–57. 10.1093/jnci/94.5.334 11880473

[13] Ellis L Canchola AJ Spiegel D Ladabaum U Haile R Gomez SL. Racial and ethnic disparities in cancer survival: the contribution of tumor, sociodemographic, institutional, and neighborhood characteristics. J Clin Oncol. (2018) 36:25–33. 10.1200/JCO.2017.74.2049 29035642 PMC5756323

[14] Samorani M Harris SL Blount LG Lu H Santoro MA. Overbooked and overlooked: machine learning and racial bias in medical appointment scheduling. Manuf Serv Oper Manag. (2021) 1:1–18. 10.1287/msom.2021.0999

[15] Sotudian S Afran A LeBedis CA Rives AF Paschalidis IC Fishman MDC. Social determinants of health and the prediction of missed breast imaging appointments. BMC Health Serv Res. (2022) 22:1454. 10.1186/s12913-022-08784-8 36451240 PMC9714014

[16] Panzone J Welch C Morgans A Bhanvadia SK Mossanen M Shenhav-Goldberg R Association of race with cancer-related financial toxicity. JCO Oncol Pract. (2022) 18:e271–83. 10.1200/OP.21.00440 34752150

[17] Han X Zhao J Zheng Z de Moor JS Virgo KS Yabroff KR. Medical financial hardship intensity and financial sacrifice associated with cancer in the United States. Cancer Epidemiol Biomarkers Prev. (2020) 29:308–17. 10.1158/1055-9965.EPI-19-0460 31941708 PMC7007367

[18] Ramsey SD Bansal A Fedorenko CR Blough DK Overstreet KA Shankaran V Financial insolvency as a risk factor for early mortality among patients with cancer. J Clin Oncol. (2016) 34:980–6. 10.1200/JCO.2015.64.6620 26811521 PMC4933128

[19] Patterson A Robinson TJ Roberts ET. Racial and ethnic disparities in insurance coverage among US adults aged 60 to 64 years. JAMA Netw Open. (2022) 5:229406. 10.1001/jamanetworkopen.2022.9406 PMC9051983 35482311

[20] SnyderRAHuCYDiBritoSRChangGJ. Association of medicaid expansion with racial disparities in cancer stage at presentation. Cancer. (2022) 128(18):3340–51. 10.1002/cncr.3434735818763

[21] Neal RD Tharmanathan P France B Din NU Cotton S Fallon-Ferguson J Is increased time to diagnosis and treatment in symptomatic cancer associated with poorer outcomes? Systematic review. Br J Cancer. (2015) 112:S92–S107. 10.1038/bjc.2015.48 25734382 PMC4385982

[22] Khorana AA Tullio K Elson P Pennell NA Grobmyer SR Kalady MF Time to initial cancer treatment in the United States and association with survival over time: an observational study. PLoS One. (2019) 14:e0213209. 10.1371/journal.pone.0213209 30822350 PMC6396925

[23] Cone EB Marchese M Paciotti M Nguyen DD Nabi J Cole AP Assessment of time-to-treatment initiation and survival in a cohort of patients with common cancers. JAMA Netw Open. (2020) 3:e2030072. 10.1001/jamanetworkopen.2020.30072 33315115 PMC7737088

[24] Wyatt RM Jones BJ Dale RG. Radiotherapy treatment delays and their influence on tumor control achieved by various fractionation schedules. Br J Radiol. (2008) 81:549–63. 10.1259/bjr/94471640 18378526

[25] Lash RS Hong AS Bell JF Reed SC Pettit N. Recognizing the emergency department’s role in oncologic care: a review of the literature on unplanned acute care. Emerg Cancer Care. (2022) 1(1):6. 10.1186/s44201-022-00007-4 35844666 PMC9200439

[26] Philpott-Morgan S Thakrar DB Symons J Ray D Ashrafian H Darzi A Characterising the nationwide burden and predictors of unkept outpatient appointments in the national health service in England: a cohort study using a machine learning approach 2021. PLoS Med. (2021) 18:e1003783. 10.1371/journal.pmed.1003783 34637437 PMC8509877

[27] Kheirkhah P Feng Q Travis LM Tavakoli-Tabasi S Sharafkhaneh A. Prevalence, predictors and economic consequences of no-shows. BMC Health Serv Res. (2015) 16:1. 10.1186/s12913-015-1243-z PMC4714455 26769153

[28] Marbouh D Khaleel I Al Shanqiti K Al Tamimi M Simsekler MCE Ellahham S Evaluating the impact of patient no-shows on service quality. Risk Manag Healthc Policy. (2020) 13:509–17. 10.2147/RMHP.S232114 32581613 PMC7280239

[29] Gier J. Missed appointments cost the U.S. Healthcare system $150B each year. Nashville, TN, United States: Healthcare Innovation (2017). Available at: https://www.hcinnovationgroup.com/clinical-it/article/13008175/missed-appointments-cost-the-us-healthcare-system-150b-each-year (Accessed January 1, 2023).

[30] Berg BP Murr M Chermak D Woodall J Pignone M Sandler RS Estimating the cost of no-shows and evaluating the effects of mitigation strategies. Med Decis Making. (2013) 33:976–85. 10.1177/0272989X13478194 23515215 PMC4153419

[31] Weinstein E Ragazzoni L Burkle F Allen M Hogan D Della Corte F. Delayed primary and specialty care: the coronavirus disease–2019 pandemic second wave. Disaster Med Publ Health Preparedness. (2020) 14:e19–21. 10.1017/dmp.2020.148 PMC7248587 32438940

[32] Cuevas AG O’Brien K. Racial centrality may be linked to mistrust in healthcare institutions for African Americans. J Health Psychol. (2019) 24:2022–30. 10.1177/1359105317715092 28810474

[33] Fillon M. Medicaid expansion increases survival for patients with cancer. CA Cancer J Clin. (2022) 72:403–502. 10.3322/caac.21751 36069380

[34] Ansell D Crispo JG Simard B Bjerre LM. Interventions to reduce wait times for primary care appointments: a systematic review. BMC Health Serv Res. (2017) 17:295. 10.1186/s12913-017-2219-y 28427444 PMC5397774

[35] Townsend M Kallogjeri D Scott-Wittenborn N Gerull K Jansen S Nussenbaum B. Multidisciplinary clinic management of head and neck cancer. JAMA Otolaryngol Head Neck Surg. (2017) 143:1213–9. 10.1001/jamaoto.2017.1855 29075744 PMC5824300

[36] Basta YL Tytgat KA Greuter HH Klinkenbijl JHG Fockens P Strikwerda J. Organizing and implementing a multidisciplinary fast track oncology clinic. Int J Qual Health Care. (2017) 29:966–71. 10.1093/intqhc/mzx143 29177408

[37] Coppa K Kim EJ Oppenheim MI Bock KR Zanos TP Hirsch JS. Application of a machine learning algorithm to develop and validate a prediction model for ambulatory non-arrivals. J Gen Intern Med. (2023) 38(10):2298–307. 10.1007/s11606-023-08065-y 36757667 PMC9910253

[38] Chong LR Tsai KT Lee LL Foo SG Chang PC. Artificial intelligence predictive analytics in the management of outpatient MRI appointment no-shows. AJR Am J Roentgenol. (2020) 215:1155–62. 10.2214/AJR.19.22594 32901567

